# Temporal changes of soil physic-chemical properties at different soil depths during larch afforestation by multivariate analysis of covariance

**DOI:** 10.1002/ece3.947

**Published:** 2014-03-05

**Authors:** Hui-Mei Wang, Wen-Jie Wang, Huanfeng Chen, Zhonghua Zhang, Zijun Mao, Yuan-Gang Zu

**Affiliations:** Key Laboratory of Forest Plant Ecology, Northeast Forestry UniversityHarbin, 150040, China

**Keywords:** Divergent changes between surface and deep soil, plant–soil interactions, soil fertility, temporal changes evaluation, vertical changes

## Abstract

Soil physic-chemical properties differ at different depths; however, differences in afforestation-induced temporal changes at different soil depths are seldom reported. By examining 19 parameters, the temporal changes and their interactions with soil depth in a large chronosequence dataset (159 plots; 636 profiles; 2544 samples) of larch plantations were checked by multivariate analysis of covariance (MANCOVA). No linear temporal changes were found in 9 parameters (N, K, N:P, available forms of N, P, K and ratios of N: available N, P: available P and K: available K), while marked linear changes were found in the rest 10 parameters. Four of them showed divergent temporal changes between surface and deep soils. At surface soils, changing rates were 262.1 g·kg^−1^·year^−1^ for SOM, 438.9 mg·g^−1^·year^−1^ for C:P, 5.3 mg·g^−1^·year^−1^ for C:K, and −3.23 mg·cm^−3^·year^−1^ for bulk density, while contrary tendencies were found in deeper soils. These divergences resulted in much moderated or no changes in the overall 80-cm soil profile. The other six parameters showed significant temporal changes for overall 0–80-cm soil profile (P: −4.10 mg·kg^−1^·year^−1^; pH: −0.0061 unit·year^−1^; C:N: 167.1 mg·g^−1^·year^−1^; K:P: 371.5 mg·g^−1^ year^−1^; N:K: −0.242 mg·g^−1^·year^−1^; EC: 0.169 *μ*S·cm^−1^·year^−1^), but without significant differences at different soil depths (*P *>* *0.05). Our findings highlight the importance of deep soils in studying physic-chemical changes of soil properties, and the temporal changes occurred in both surface and deep soils should be fully considered for forest management and soil nutrient balance.

## Introduction

Many forests are planted on degraded or abandoned farmlands, for use as agricultural protection forests, aimed at timber production, ecological protection, and degraded land rehabilitation (FAO 2010; Wang et al. 2011). In addition to the significance of aboveground biomass accumulation, there are increasing concerns regarding soil organic matter (SOM) dynamics and possible nutrient limitation as well as soil degradation in plantation forests. Tree roots are generally longer than those of herbs and other crops (Stone and Kalisz 1991; Canadell et al. 1996). Consequently, more nutrients could be exploited from deep soils together with more labile carbohydrates from root exudations, and possible accelerated decomposition from priming effect (Fontaine et al. 2007). Compared with surface soil, the deep-root features and close-link between SOM and nutrient cycles request a full-check on the temporal changes of different soil physic-chemical properties in deep soils, which is crucial for a comprehensive understanding of afforestation-induced soil changes in physical feature, soil fertility, and stoichiometric composition.

Soil degradation including nutrient depletion, soil acidification, and physical compaction may constrain tree growth of larch plantation in a long term (Pan and Wang 1997; Liu et al. 1998; Chen and Xiao 2006). Marked temporal changes in different soil parameters of SOM, N, P, K, available N, available P, available K, or soil acidity are indicators for soil degradation from long-term afforestation (Yang et al. 2008; Wang et al. 2011). Forest growth may also result in stoichiometric compositional changes (e.g., C:N increases) (Pan and Wang 1997; Liu et al. 1998; Wang et al. 2011). However, few studies have examined the annual changing rates of these soil properties and their interaction with soil depths (Wang et al. 2011). The avoidance of forest soil degradation as well as nutrient budget estimation needs the supports from such spatial–temporal data of soils (Liu et al. 1998; Cleveland and Liptzin 2007; Manzoni et al. 2010; Huang et al. 2013).

Identification of forest growth on soil properties (temporal changes) becomes difficult owing to the large soil heterogeneity at different soil depths. Regression at different soil layers and confidence intervals (Wang et al. 2011), and Bonferroni correction and power analysis (Guo et al. 2004; Wang et al. 2011) have been used to improve the statistical power. MANCOVA (multivariate analysis of covariance),a general linear model which blends analysis of variance and linear regression, has been used to linearly exclude effects from different covariates for identifying the treatment effects in soil sciences (Zammit and Zedler 1988; Torn and Harte 1996; Yamashita et al. 2008). Together with a large chronosequence dataset, MANCOVA may favor the identification of the linear temporal changes in different soil horizons (Engqvist 2005; Green and Salkind 2011; Fox et al. 2013). These data are important for highlighting the importance of deep soils in nutrient dynamics and soil physiochemical changes for deep-root forest ecosystem (Rumpel and Kögel-Knabner 2011; Wang et al. 2011) as well as proposals of effective methods for soil degradation avoidance (Liu et al. 1998).

In the present study, we evaluated temporal variations in SOM, N, P, K, available N, available P, available K, pH, EC, and bulk density in surface and deep soils of typical larch plantation forests in northeastern China. We based our study on the following hypotheses: besides surface soil, larch plantations can induce marked temporal changes of different soil properties in deep soils, and these should be considered in afforestation practices and forest managements.

## Materials and Methods

### Study sites and sample collection

**Figure 1:**
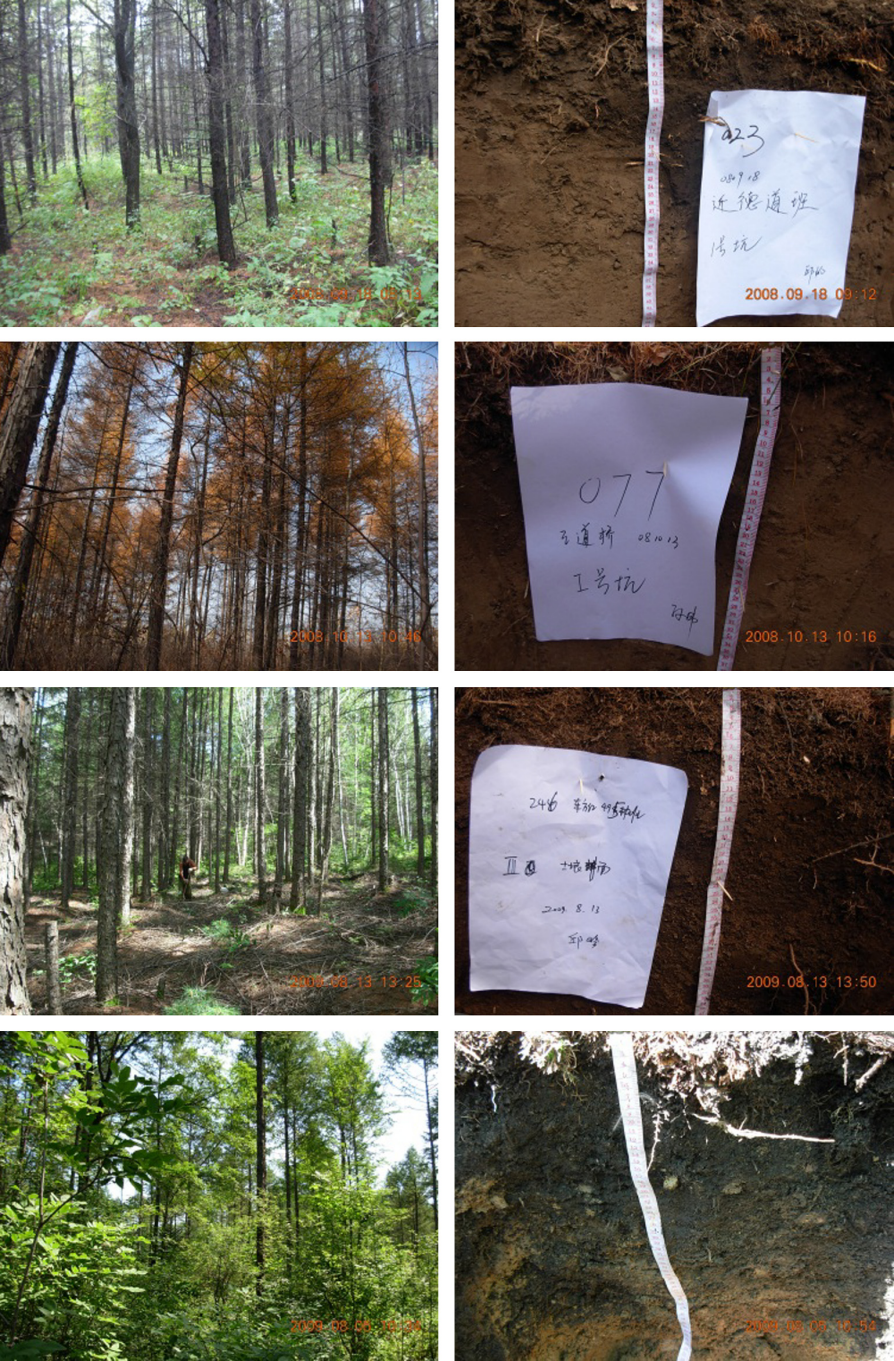
Larch plantations in northeastern China and soil profiles used in this paper.

The study sites were located centrally within *Larix gmelinii* Rupr. plantations in northeastern China. The locations of the 159 plots ranged from 45°20′N to 47°00′N and from 127°33′E to 129°10′E. In addition to the stand age, topographic position, slope, climatic difference, elevation, cultivation history, plantation managements, and soil origin may influence SOM and soil fertility. To minimize the influences of these nonchronosequence sources, all of the plots were restricted to a region of typical flat plain with shallow hills, and a similar elevation of approximately 280 m (SD 43 m) and slope of <20% (Fig. 1). This region has a continental monsoon climate, with an average annual temperature of 0.3–2.6°C and annual precipitation of 676–724 mm. All of the study plots were historically farmland, producing soybean or corn over the short (as short as several years) or long term (decades). All of the plantation forests were national forests from government-administered forest farms and were subject to similar tending and thinning forest management practices. The soil in the study region is generally a typical dark-brown forest soil (with some regions characterized as lessive dark-brown forest soil) (Fig. 1), which originated from forestlands of the climatic climax of Korean pine-broadleaved mixed forests (Wang et al. 2011).

In each plot, we surveyed a 20 m × 20 m quadrat and excavated 4 soil profiles (2 m in length, 1 m in width, and 0.8 m in depth). After exclusion of the A_0_ layer (recognizable litter at the soil surface), we sampled 100 cm^3^ of soil from each of the 4 soil profiles, at depths of 0–20 cm, 20–40 cm, 40–60 cm, and 60–80 cm. Therefore, in each plot, we collected 16 samples (4 samples per soil profile and 4 soil profiles per plot). Thus, in total, we collected 2544 samples (16 samples per plot and 159 plots) from 636 soil profiles (4 soil profiles per plot and 159 plots).

We determined the forest age using an increment borer (Zhonglinweiye, Beijing, China), with a minimum of five replicates. The maximum and minimum forest ages were 52 years and 0 years (just before return from farmland to forest), respectively. The maximum diameter at breast height (DBH) and the maximum tree height were 39 cm and 30 m, respectively. Forest age was significantly related to DBH and tree height (*R*^2^ > 0.7, *P *<* *0.0001). The average annual increment of DBH and the average increase in tree height were 0.62 cm and 0.55 m·year^−1^, respectively. The growth of larch plantations induced a significant increase in aboveground and belowground biomass. The average branch biomass increase was 0.32 ton·hm^−2^·year^−1^; the average leaf biomass increase was 0.08 ton·hm^−2^·year^−1^; the average stem biomass increase was 4.53 ton·hm^−2^·year^−1^; and the average root biomass increase was 1.35 ton·hm^−2^·year^−1^. Litter mass in the forest floor was significantly correlated with tree height; a 1-m rise in tree height was equivalent to an increase in litter mass of 76.27 g·m^−2^ (Wang et al. 2012).

### Determination of soil parameters

After carefully removing roots from the soil samples, we prepared soil samples with grain sizes of <0.25 mm for future analysis, using a powerful grinder. We measured SOM using the heated dichromate titration method and calculated the change from soil organic carbon (SOC) to SOM by multiplying by 1.724 (Bao 2000); for details, see Wang et al. (2011).

We determined soil total nitrogen (N) using the semimicro-Kjeldahl procedure (Bao 2000) and measured soil available nitrogen (available N) using the NaOH-hydrolyzing-NH_3_-diffusing-H_3_BO_3_-absorption method (Bao 2000). Readily hydrolytic nitrogen (ammonium N, nitrate N, amino acid, amide, and readily hydrolyzable protein) in the soil samples was transformed to ammonia by the addition of NaOH solution to the outer ring of the Conway dish. The ammonia gas was diffused and absorbed by H_3_BO_3_ solution in the inner ring of the Conway dish. Titration with H_2_SO_4_ solution was used to calculate the available N in soil samples.

We measured soil total phosphorus (P) using the NaOH fusion–Mo-Sb antispectrophotometric method (Bao 2000). The soil samples and NaOH were fused in nickel crucibles at 720°C in a muffle furnace, and the digested solution was transferred to a 50-mL volumetric flask for measurement of soil total P. Double-acid-extractable P (available P) was extracted using HCl–H_2_SO_4_ solution. A fixed volume of this solution (5 mL) was mixed with Mo–Sb chromogenic agent (a solution of l-ascorbic acid and H_2_SO_4_–(NH_4_)_6_Mo_7_O_24_–K(SbO)C_4_H_4_O_6_), and a spectrophotometer was used to measure the absorption spectrophotometry at 700 nm (Unico 2100, Shanghai, China). The readings were used to recalculate the content of soil available P from the calibration curve.

We measured soil total potassium (K) using the NaOH fusion–flame spectrometric method (Bao 2000). The digested solution described above for soil total P measurement was also used for soil total K measurement. Soil available potassium (available K) was extracted using 1 N NH_4_OAc solution (5 g of soil in a 50-mL solution; shaken for 30 min) (Bao 2000). The content of K was determined using a flame atomic absorption spectrometer (novAA350, Jena, Germany), and the readings were used to recalculate the content of soil available K from the calibration curve.

The soil bulk density was determined by placing 400-cm^3^ samples in a cloth bag and airing them in a dry ventilated room until they reached constant weight. The pH and electrical conductance (EC) of the soil solution (one part soil to five parts water) were measured using an acidity meter (Sartorius PT-21, Shanghai, China) and electrical conductance meter (Leici, Shanghai, China), respectively.

### Data analysis

Nineteen soil parameters of SOM, N, P, K, available N, available P, and available K, pH, EC, bulk density and the ratios of C:N, C:P, C:K,N:P, N:K, K:P, N:available N, P:available P, K:available K were tested in this paper. The temporal changing rates at different soil depths were statistically analyzed by MANCOVA with soil depth as the independent factor and forest age as the covariate. Multivariate tests were firstly used to check the influences of soil depth, tree age on the ecosystem properties, and the parameter of Pillai's trace was computed for this analysis (Fox et al. 2013). A significant interaction indicates that the temporal changing rate (slope) of the tested soil properties significantly differs at different soil layers, and parameter estimation in the option menu of MANCOVA was selected for calculation of the slopes. We performed all analyses with SPSS software (SPSS 17.0, Chicago, IL).

## Results

### Multivariate tests by soil depth, tree age, and their interaction

As shown in Table 1, multivariate test showed that Pillai's trace values for soil depth, tree age and depth*age interaction were statistically significant (*P* < 0.01). All these statistics showed that soil depth and tree age could significantly affect the ecosystem properties as a whole (*P *<* *0.001). Moreover, significant interactions between soil depth and tree age indicate that the temporal changes of the ecosystem properties are also depend on soil depth (*P *<* *0.005). Univariate ANCOVA following the MANCOVA results as shown in Table 2 was used to dissect the patterns (vertical pattern and temporal changing pattern) specific to each variable.

**Table 1:** Multivariate tests for the MANCOVA with soil depth and covariate of tree age.

Effect	Pillai's trace value	*F*	Hypothesis df	Error df	Significance
Soil depth	0.335	3.496	57	1584	0.000
Tree age	0.140	4.490	19	526	0.000
Depth × age interaction	0.167	1.641	57	1584	0.002

**Table 2:** Results of MANCOVA with soil depth as the independent factor and forest age as the covariate. All results for interactions were from homogeneity of regression slopes.

No	Parameter	Soil depth (Factor)		Forest age (Covariate)		Interaction	
		*F*	Significance	*F*	Significance	*F*	Significance
Heterogeneous slopes							
1	SOM content (g kg^−1^)	99.77	0.000	0.002	0.961	3.720	0.011
2	C:P ratio	125.3	0.000	21.75	0.000	12.46	0.000
3	C:K ratio	103.8	0.000	0.002	0.966	4.37	0.005
4	Bulk density (g cm^−3^)	2736	0.000	0.14	0.710	6.82	0.000
Significant homogeneous slopes							
5	P content (g kg^−1^)	169.6	0.000	8.931	0.003	0.434	0.729
6	C:N ratio	1.093	0.352	20.59	0.000	0.586	0.624
7	K:P ratio	86.0	0.000	17.89	0.000	0.539	0.656
8	N:K ratio	103.0	0.000	4.475	0.035	0.163	0.921
9	PH	0.880	0.451	17.16	0.000	1.066	0.363
10	EC (*μ*S cm^−1^)	138.4	0.000	12.49	0.000	0.381	0.767
Nonsignificant homogeneous slopes							
11	N content (g kg^−1^)	101.1	0.000	3.471	0.063	0.157	0.925
12	K content (g kg^−1^)	932.3	0.000	0.154	0.695	0.076	0.973
13	N:P ratio	168.5	0.000	0.104	0.747	1.160	0.324
14	Available K (mg kg^−1^)	31.51	0.000	1.871	0.172	0.166	0.919
15	Available P (mg kg^−1^)	22.69	0.000	1.096	0.296	0.118	0.950
16	Available N (mg kg^−1^)	73.11	0.000	0.972	0.325	0.247	0.863
17	P:available P ratio	3.521	0.015	1.642	0.201	0.852	0.466
18	K:available K ratio	3.81	0.005	1.126	0.289	0.239	0.869
19	N:available N ratio	37.69	0.000	1.761	0.185	0.362	0.780

### Interaction between forest age and soil depth: contrary temporal changes between surface and deep soils

According to Table 2, the homogeneity test of regression slopes showed marked different temporal changes at different soil layers in 4 parameters of SOM, C:P,C:K, and bulk density. Linear slopes and their statistics from MANCOVA parameter estimation are listed in Table 3.

**Table 3:** Comparison of the significant changing rates (slopes) and other related parameters derived from the homogeneity test of regression slopes and the analysis of covariance (MANCOVA). Bold font indicates the changing rates with a significance level *P *<* *0.05.

Variables	Soil layer (cm)	Slope	Standard error	*t*-value	Significance	95% confidence interval	
						Lower bound	Upper bound
Different changing rates between deep and surface soils (Heterogeneous slopes)							
SOM (mg·kg^−1^·year^−1^)	0–20*	**262.1**	**125.4**	**2.091**	**0.037**	**15.9**	**508.4**
	20–40*	−36.2	125.4	−0.288	0.77	−282.6	210.2
	40–60*	−89.5	129.8	−0.690	0.49	−344.5	165.5
	60–80*	−123.9	125.7	−0.986	0.33	−370.8	123.0
	Overall profile	4.91	63.43	0.077	0.938	−119.7	129.5
C:P (mg·g^−1^·year^−1^)	0–20*	**438.9**	**60.9**	**7.208**	**0.000**	**319.3**	**558.5**
	**20–40**	**137.2**	**60.9**	**2.253**	**0.025**	**17.6**	**256.9**
	40–60*	69.1	63.0	1.096	0.274	−54.7	192.9
	60–80*	−71.8	61.0	−1.175	0.240	−191.7	48.2
	**Overall profile**	**144.9**	**31.7**	**4.575**	**0.000**	**82.7**	**207.2**
C:K (mg·g^−1^·year^−1^)	0–20*	**5.3**	**2.7**	**1.994**	**0.047**	**0.1**	**10.5**
	20–40*	−0.9	2.7	−0.345	0.730	−6.1	4.3
	40–60*	−2.3	2.8	−0.830	0.407	−7.7	3.1
	60–80*	−2.3	2.7	−0.873	0.383	−7.6	2.9
	Overall profile	−0.015	1.3	−0.011	0.991	−2.7	2.6
Bulk density (mg·cm^−3^·year^−1^)	0–20*	−**3.23**	**0.94**	−**3.448**	**0.001**	−**5.07**	−**1.39**
	20–40*	0.06	0.94	0.059	0.953	−1.78	1.89
	40–60*	1.70	0.94	1.813	0.070	−0.14	3.54
	60–80*	**2.18**	**0.94**	**2.329**	**0.020**	**0.34**	**4.02**
	Overall profile	0.18	0.47	0.371	0.71	−0.76	1.11
Similar changing rate overall soil profile (Homogeneous slopes)							
P (mg·kg^−1^·year^−1^)	0–20*	−**5.6**	**2.7**	−**2.07**	**0.039**	−**10.9**	−**0.3**
	20–40*	−**5.3**	**2.7**	−**1.96**	**0.051**	−**10.7**	**0.0**
	40–60*	−3.7	2.8	−1.33	0.183	−9.3	1.8
	60–80*	−1.7	2.7	−0.63	0.531	−7.1	3.6
	Overall profile	−**4.10**	**1.37**	−**3.00**	**0.003**	−**6.8**	−**1.4**
C:N (mg·g^−1^·year^−1^)	0–20*	**249.7**	**72.8**	**3.431**	**0.001**	**106.7**	**392.7**
	20–40*	136.6	72.8	1.876	0.061	−6.4	279.7
	40–60*	**149.2**	**75.4**	**1.980**	**0.048**	**1.2**	**297.3**
	60–80*	131.4	73.0	1.800	0.072	−12.0	274.7
	Overall profile	**167.1**	**36.7**	**4.554**	**0.000**	**95.0**	**239.2**
K:P (mg·g^−1^·year^−1^)	0–20*	259.7	175.4	1.480	0.139	−84.9	604.2
	20–40*	**368.9**	**175.5**	**2.102**	**0.036**	**24.1**	**713.7**
	40–60*	**560.7**	**181.6**	**3.087**	**0.002**	**203.9**	**917.4**
	60–80*	309.1	175.9	1.757	0.079	−36.4	654.6
	Overall profile	**371.5**	**88.4**	**4.202**	**0.000**	**197.8**	**545.2**
N:K (mg·g^−1^·year^−1^)	0–20*	−0.27	0.23	−1.188	0.235	−0.71	0.18
	20–40*	−0.35	0.23	−1.532	0.126	−0.79	0.10
	40–60*	−0.22	0.23	−0.952	0.341	−0.68	0.24
	60–80*	−0.13	0.23	−0.564	0.573	−0.57	0.32
	Overall profile	−**0.242**	**0.114**	−**2.125**	**0.034**	−**0.466**	−**0.018**
EC (*μ*S·cm^−1^·year^−1^)	0–20*	**0.221**	**0.095**	**2.325**	**0.020**	**0.034**	**0.408**
	20–40*	0.088	0.095	0.928	0.354	−0.099	0.275
	40–60*	**0.203**	**0.098**	**2.065**	**0.039**	**0.010**	**0.397**
	60–80*	0.166	0.095	1.741	0.082	−0.021	0.353
	Overall profile	**0.169**	**0.048**	**3.530**	**0.000**	**0.075**	**0.263**
pH (unit year^−1^)	0–20*	−2.05E−03	2.92E-03	−0.70	0.483	−7.8E-03	3.68E-03
	20–40*	−**5.57E-03**	**2.9E-03**	−**1.91**	**0.057**	−**1.1E-02**	**1.6E-04**
	40–60*	−**8.01E-03**	**3.0E-03**	−**2.65**	**0.008**	−**1.4E-02**	−**2.1E-03**
	60–80*	−**8.77E-03**	**2.9E-03**	−**3.00**	**0.003**	−**1.5E-02**	−**3.0E-03**
	Overall profile	−**6.06E-03**	**1.5E-03**	−**4.12**	**0.000**	−**9.0E-03**	−**3.2E-03**
No changes overall soil profile (nonsignificant homogeneous slopes at different soil depth)							
N (mg·kg^−1^·year^−1^)	Overall profile	−5.69	3.05	−1.867	0.062	−11.7	0.3
K (mg·kg^−1^·year^−1^)	Overall profile	7.22	18.84	0.383	0.702	−29.8	44.2
N:P (mg·g^−1^·year^−1^)	Overall profile	0.797	2.3	0.345	0.730	−3.7	5.3
Available K (mg·kg^−1^·year^−1^)	Overall profile	−215.5	156.1	−1.380	0.168	−522.2	91.2
Available P (mg·kg^−1^·year^−1^)	Overall profile	−73.4	70.9	−1.036	0.301	−212.6	65.8
Available N (mg·kg^−1^·year^−1^)	Overall profile	−172.3	173.9	−0.991	0.322	−514.0	169.3
P:available P (g·g^−1^·year^−1^)	Overall profile	4.28	3.30	1.297	0.195	−2.20	10.75
K:available K (g·g^−1^·year^−1^)	Overall profile	247.1	235.4	1.049	0.294	−215.4	709.6
N:available N (g·g^−1^·year^−1^)	Overall profile	−0.14	0.10	−1.336	0.182	−0.34	0.06

* Indicates that the results were from the test of homogeneity of regression slopes, while all others for overall profile were from MANCOVA.

For SOM, marked positive slopes (262.1 ± 125.4 mg·kg^−1^·year^−1^) were found in 0–20-cm soil (*P *<* *0.05), while those in deeper soils were negative (−36.2 to −123.9 mg·kg^−1^·year^−1^) without statistical significance (*P *>* *0.05) (Table 3). These contrary changes between surface and deep soils resulted in the observation of no changes in the overall 0–80-cm soils (slope = 4.91 mg·kg^−1^·year^−1^, *P *=* *0.938) (Table 3).

For C:P ratio, marked temporal increases in 0–20-cm soil (438.9 ± 60.9 mg·g^−1^·year^−1^) (*P *=* *0.000) and in 20–40-cm soil (137.2 ± 60.9 mg·g^−1^·year^−1^) (*P *=* *0.025) were observed, while decreasing tendency in deeper soil (60–80-cm soil) was found (−71.8 mg·g^−1^·year^−1^) (*P *=* *0.240). These contrary temporal changes strongly moderated the temporal changes of whole 0–80-cm soil, 144.9 ± 31.7 mg·g^−1^·year^−1^ (*P *=* *0.000). This rate was only one-third of that observed in 0–20-cm soil (Table 3).

For C:K ratio, a marked temporal increase (5.3 ± 2.7 mg·g^−1^·year^−1^) was observed in 0–20-cm soil, while decreasing tendencies were observed in deeper soils (Table 3). These contrary changing tendencies resulted in no changes for overall 0–80-cm soil (slope = −0.015, *P *=* *0.991) (Table 3).

For soil bulk density, a marked decrease (−3.23 ± 0.94 mg·cm^−3^·year^−1^) in 0–20-cm soil but increasing tendencies in deeper soils were generally observed (Table 3). The increasing rate in 60–80-cm soil was 2.18 ± 0.94 mg·cm^−3^·year^−1^ (*P *=* *0.020). These contrary changes resulted in no changes in whole-soil profiles (slope = 0.18 ± 0.47 mg·cm^−3^·year^−1^, *P *=* *0.71) (Table 3).

### Significant influences from covariate of forest age in MANCOVA: consistent temporal changes in overall soil profile

In all the 15 soil parameters with homogeneous regression slopes (*P *>* *0.05 for interaction of soil depth and forest age, Table 2), significant influences from covariate forest age were observed on 6 parameters: P content, C:N ratio, K:P ratio, N:K ratio, EC, and pH (*P *<* *0.05) (Table 2). The overall regression slopes for 0–80-cm soil profile were computed by parameter estimation in performing MANCOVA, while regression slopes at different soil layer were computed in homogeneity test of regression slopes (Table 3).

In the case of P, depletion for overall soil profile was −4.10 ± 1.37 mg·kg^−1^·year^−1^, *P *=* *0.003), and 95% confidence interval ranged from −6.8 to −1.4·mg kg^−1^·year^−1^. Similar temporal changing rates (−5.6 in 0–20-cm to −5.3 mg·kg^−1^·year^−1^ in 20–40-cm soil) were found in the regression slopes at different soil layers (Table 3).

In the case of C:N, temporal increasing rate for overall 0–80-cm soils was 167.1 ± 36.7 mg·g^−1^·year^−1^ (*P *=* *0.000), and 95% confidence interval ranged from 95.0 to 239.2 mg·g^−1^·year^−1^. Data of temporal changes at different soil layers showed 2 significant regressions at 0–20 cm, 60–80-cm soils, and the changing rates ranged from 149.2 to 249.7 mg·g^−1^·year^−1^ (Table 3).

In the case of K:P ratio, temporal increasing rate for overall soil profile was 371.5 ± 88.4 mg·g^−1^·year^−1^, and 95% confidence interval ranged from 197.8 to 545.2 mg·g^−1^·year^−1^ (Table 3). Two of the 4 soil layers showed marked regression slopes, and range for these temporal changing rates (368.9 to 560.7 mg·g^−1^·year^−1^) was within those (95% confidence interval) observed in overall soil profile (Table 3).

In the case of N:K ratio, temporal increasing rate for overall soil profile was −0.242 ± 0.114 mg·g^−1^·year^−1^, and 95% confidence interval ranged from −0.466 to −0.018 mg·g^−1^·year^−1^ (Table 3). None of the four soil layers showed marked regression slopes (Table 3).

In the case of EC, temporal increasing rate was 0.169 ± 0.048 *μ*S·cm^−1^·year^−1^, and 95% confidence interval ranged from 0.075 to 0.263 *μ*S cm^−1^·year^−1^(Table 3). For different soil layers, 2 significant regressions were observed, and the temporal changing rates for these soil layers ranged from 0.203 to 0.221 *μ*S·cm^−1^·year^−1^ (Table 3).

The overall decreasing rate for pH was 6.06 × 10^−3^ ± 1.5 × 10^−3^ unit year^−1^, and the 95% confidence interval ranged from 3.2 × 10^−3^ to 9.0 × 10^−3^ unit year^−1^(Table 3). All regression slopes at different soil layers were statistically significant (*P *<* *0.05) except surface 20-cm soil. The decreasing rates ranged from 5.57 × 10^−3^ to 8.77 × 10^−3^ unit year^−1^ (Table 3).

### Nonsignificant influences from covariate of forest age in MANCOVA: no changes through overall soil profiles during forest development

Nine parameters showed nonsignificant temporal changes in overall soil profile. These parameters were N, K, N:P, available forms of N, P, K and ratios of N: available N, P: available P and K: available K (Table 2). The overall regression slopes were also computed by the parameter estimation in MANCOVA (Table 3).

The sign (plus or negative) of the slopes could show the changing tendencies of variable soil properties, although all these were not statistical significant (*P *>* *0.05). There were decreasing tendencies in five parameters (N, available N, available P, available K, and N:available N), while accumulation tendencies were found in the other four parameters (K, N:P, P: available P, and K: available K) (Table 3).

## Discussion

### Deep soil importance in studying soil dynamics

Surface soil is generally believed to be the most active layer in terms of fertility-related parameters, while the role of deep soil is seldom highlighted (Rumpel and Kögel-Knabner 2011). In fact, most nutrients for larch plantations in northeastern China were stored in surface soil, while concentration of some others (e.g., K) was higher in deep soils (Table 4). These nutritional vertical differences caused the observation of much higher values of C:P, N:P, N:K, C:K in surface soil, but much higher value of K:P in deep soil (Table 4). Thus, surface soil is undisputed important for storage of most nutrients and supply of biomass growth. However, determination of temporal changes of variable soil properties in surface soil alone may produce bias-estimations, and our study strongly supports this conclusion (Tables 2 and 3).

An old assumption for deep soil is the relative inert feature for period of years or decades of years, and many previous studies focused on surface soil alone based on this assumption. Within 50-year afforestation in abandoned farmlands in our present study, over half parameters (10 of 19 parameters) showed evident temporal changes as deep as 80-cm soils (Table 2), indicating this assumption is invalid for deep-root vegetation of forests. Moreover, these temporal changes differed from different soil parameters, and with significant interaction with soil depth. Some of them (P, C:N, K:P, N:K, EC, and pH) showed consistent temporal changes in overall 0-80-cm soil, while the other 4 soil parameters (SOM, C:P, C:K,bulk density) showed contrary temporal changes between surface and deep soil layers (*P *<* *0.05) (Tables 2 and 3). Compared with the surface soil alone, these contrary temporal changes could strongly moderate the changing rate of the tested parameters on the whole-soil profile. In the case of similar changing rates between surface and deep soils, estimation of soil nutrient changing rates in concentration can be derived by surface soil alone (Table 3). However, nutrient consumption from soil can obviously be underestimated if calculation of nutrient budget based on surface soil alone, and temporal changes in nutrient storage at different soil layers are needed for characterizing this underestimation. The importance of deep soil has recently highlighted (Rumpel and Kögel-Knabner 2011). In the case of studying temporal changes of different soil properties in forest ecosystem, deep soils should be fully considered and most possible parameters include SOM, P, EC, pH, bulk density, and stoichiometric ratios C, N, P, and K (Tables 2 and 3). The finding in this paper is a supplement of previous studies.

Similar to this study, afforestation-induced marked changes in deep soils have previously been demonstrated in variable soil properties. According to a long-term plot study, the SOC content increased from 5.5 g·kg^−1^ to 6.7 g·kg^−1^ in the surface layer (7.5 cm) of mineral soils, but decreased from 3.8 g·kg^−1^ to 2.5 g·kg^−1^ in deeper layers (15–35 cm) (Richter et al. 1999). Chapela et al. (2001) demonstrated that, after 12 years of pine tree introduction to grasslands, the SOC content of the 0–10-cm surface soil remained almost unchanged (78 g·kg^−1^), whereas that of the 10–20-cm and 20–30-cm soil layers decreased by 30% and 44%, respectively. In northeastern China, a 25-year long-term fixed-plot study also revealed a slight increase in N storage (from 0.32 kg·m^−2^ to 0.33 kg·m^−2^) in the surface soil, but a marked decrease (from 0.11–0.16 kg·m^−2^ to 0.06–0.11 kg·m^−2^) at a depth of 20–60 cm (Chen 1986; Wang et al. 2011). For both surface and deep soils, soil acidification was observed in larch plantations (Pan and Wang 1997; Sun et al. 1997; Chen and Xiao 2006). Possible depletion of soil nutrient, nutrient redistribution on soil profile, SOC accumulation as well as fast turnover rate in deep soil were also reported (Franchini et al. 2004; Fontaine et al. 2007; Rumpel and Kögel-Knabner 2011). Our study provides additional data regarding temporal change variations of variable soil properties across the whole-soil profile.

### Soil degradation during larch afforestation: implication for afforestation practices

Compared with surface soil, either contrary or consistent temporal changes in deep soils (Tables 2 and 3) indicate the importance of deep soil for maintaining soil sustainability for above-ground productivity and their C sequestration capacity (Chapela et al. 2001; Mack et al. 2004; Rumpel and Kögel-Knabner 2011). Our present data highlighted that the soil degradation mainly observed in pH, EC, bulk density, and P during larch afforestation at degraded farmland. Although soil became lighter and loosening with more SOM at surface soil, heavier and compacted soil with increasing bulk density and decreasing SOM were generally observed in deeper soil >20-cm soils. Besides surface soil, soil acidification with increasing EC and evident P depletion was observed in deep soils. Moreover, alteration of the soil stoichiometry (i.e., steady increase in C:N and K:P ratio in overall soil profile, or contrary temporal changes in C:P and C:K) was also a feature of the soil degradation during larch afforestation (Table 3). Furthermore, the soil degradation in this paper is assumed as linear changes with forest age (Wang et al. 2011). Nonlinear changes during forest development were also reported (Covington 1981).Thus, some nonlinear soil degradation processes are overlooked in this paper and more detail analysis is needed in the future.

These larch plantation-induced soil degradations should be considered in afforestation practices. As an important and widespread global species (Gower and Richards 1990), larch plantation is also an important fast-growing forest with a productivity approximately 17.30 ton·ha^−1^·year^−1^ in northeastern China (Wang et al. 2005). The poor quality of larch litter, because of very slow decomposition and accumulation in forest floor, was a key factor contributing to soil degradation in pure larch plantations (Liu et al. 1998). The progressive accumulation of litter on the forest floor acts as a heat-isolating layer, thereby inhibiting thermal conduction, decreasing soil temperature, and limiting soil microbial activities (Liu et al. 1998; Wang et al. 2012). Thus, acceleration of nutrient cycling in the organic materials of forest floors is an important step to securing soil nutrient sustainability of larch plantations. An effective, low-cost method is to develop mixed stands, either by creating a mixture with broad-leaved trees or by introducing understory vegetation (herbs and shrubs). Nutrient mineralization will be accelerated over twofold in mixed forest (0.9–2.4 years) compared with pure larch plantation (4.4 years) (Liu et al. 1998).

## Appendix 1

**Table A1:** Vertical differences of different soil parameters in different soil depths. Marginal means and standard error were estimated by soil depth as main effect in MANCOVA and homogeneity test of regression slopes.

Variables	Soil layer (cm)	Mean	Standard error	Variables	Mean	Standard error
Decreases with soil depth				P:available P	0.217a	0.077
SOM (g kg^−1^)*	0–20	55.885a	1.411		0.391a	0.077
	20–40	27.893b	1.411		0.104a	0.078
	40–60	16.599c	1.416		0.069b	0.077
	60–80	11.020d	1.416	Increases with soil depth		
N (g kg^−1^)	0–20	2.428a	0.071	K (g kg^−1^)	26.639a	0.438
	20–40	1.250b	0.071		28.194a	0.442
	40–60	0.702c	0.072		30.327b	0.445
	60–80	0.491c	0.071		31.728b	0.440
P (g kg^−1^)	0–20	1.039a	0.032	Bulk density (g cm^−3^)*	1.099a	0.013
	20–40	0.819b	0.032		1.294b	0.013
	40–60	0.669c	0.032		1.418c	0.013
	60–80	0.605c	0.032		1.477d	0.013
Available N (mg kg^−1^)	0–20	123.459a	4.035	Available P (mg kg^−1^)	8.771a	1.646
	20–40	58.249b	4.064		9.353a	1.658
	40–60	34.401c	4.094		17.135b	1.670
	60–80	24.928c	4.050		21.638b	1.652
Available K (mg kg^−1^)	0–20	60.998a	3.628	K:P	31.788a	2.068
	20–40	34.391b	3.654		43.507b	2.083
	40–60	23.690b	3.681		56.481c	2.099
	60–80	24.766b	3.641		62.446c	2.076
C:P*	0–20	33.488a	0.679	N:available N	0.024a	0.002
	20–40	20.501b	0.679		0.028a	0.002
	40–60	14.528c	0.681		0.029a	0.002
	60–80	10.266d	0.681		0.033b	0.002
N:K	0–20	0.092a	0.003	K:available K	5.829a	5.466
	20–40	0.044b	0.003		31.829b	5.505
	40–60	0.023c	0.003		30.569b	5.546
	60–80	0.016c	0.003		39.535b	5.485
C:K*	0–20	1.233a	0.031	No changes with soil depth		
	20–40	0.578b	0.031	C/N	16.106a	0.852
	40–60	0.331c	0.031		14.563a	0.858
	60–80	0.215d	0.031		16.690a	0.864
N:P	0–20	2.340a	0.054		16.021a	0.855
	20–40	1.542b	0.054	pH	5.652a	0.034
	40–60	1.019c	0.054		5.674a	0.034
	60–80	0.834c	0.054		5.687a	0.035
EC (μS cm^−1^)	0–20	42.847a	1.116		5.731a	0.034
	20–40	31.055b	1.124			
	40–60	26.444c	1.132			
	60–80	24.830c	1.120			

* Indicates that covariates appearing in the model are evaluated at age = 22.83 (homogeneity test of regression slope), while all others are evaluated at age = 24.12 (MANCOVA).

Different letter for the same parameter at different soil depths indicates significantly vertical variations in the studied parameters between the two soil depths (*P* < 0.05), while the same letter indicates no differences existed (*P* > 0.05).
